# The association between lymph node status and the tumor size in breast cancer – results from the Danish Breast Cancer Group (DBCG)

**DOI:** 10.2340/1651-226X.2025.43380

**Published:** 2025-08-05

**Authors:** Tanja L. Fris, Marianne D. Lautrup, Peer M. Christiansen

**Affiliations:** Department of Plastic and Breast Surgery, Aarhus University Hospital, Aarhus, Denmark

**Keywords:** Breast cancer, lymphatic metastases, subtypes, algorithm

## Abstract

**Background and purpose:**

The association between the tumor size and the risk of lymph node metastasis (LNM) is well known. The purpose of this study is to describe a new model for predicting the occurrence of LNM at an earlier time for breast cancer patients where at a given time this association is known.

**Patient/material and methods:**

The subjects studied were 59,400 breast cancer patients treated in the period 1995–2012 and registered in the Danish Breast Cancer Group (DBCG) database. Data included age, year of treatment, menopausal status, tumor size, lymph node status, localization, focality, histological type, grade, estrogen receptor (ER), HER2 status, lympho-vascular invasion (LVI), and type of surgery. Univariate and multivariate analyses were made.

**Results:**

46% of patients presented with LNM. The occurrence increased with increasing tumor size. HER2 positive tumors had more LNM 56.9% versus 44.7% (*p* < 0.001) (odds ratio [OR] 1.17 [95% confidence interval, CI 1.09–1.26]) and mostly pronounced in relation to ER negative tumors (*p* < 0.001). ER negative/HER2 negative tumors had lower risk of LNM (OR 0.57 [95% CI 0.52–0.63]). Central tumors and tumors in the lower lateral quadrant were more often node positive. LVI showed increased odds for LNM (OR 5.16 [95% CI 4.84–5.52]).

**Interpretation:**

Increasing tumor size is the only time-dependent risk of LNM. HER2 positive tumors had an increased risk of LNM, and ER negative/HER2 negative tumors had a decreased risk of LNM. LVI was associated with substantial increased risk of LNM. The knowledge of breast cancer patient and tumor characteristics at a given time may predict stage of cancer at an earlier time.

## Introduction

Lymph node status is a major determinant for the treatment and prognosis in breast cancer. The incidence of lymph node metastases (LNMs) is related to the characteristics of the breast tumor and is described in several studies [1–5]. Especially, the association between the tumor size and the risk of LNM is well documented [6, 7]. To our knowledge, no publication concerning prediction of stage, if diagnosed earlier, exists.

In a previous study by The Danish Breast Cancer Group (DBCG) [8], it was shown that LNM occurs less frequently when the tumor is hormone-receptor negative compared to hormone-receptor positive tumors. Furthermore, it was shown that there is a higher incidence of LNM in HER2 positive tumors. The association between the woman’s age and the risk of LNM has also been described. Greer et al. [9] found a diminishing rate of LNM up till the age of 75, and after that the rate increased.

Breast cancer is a very complex disease presenting different tumor types and subtypes with a variable growth potential and variability in the incidence of LNMs. Models have been developed for the estimation of the growth potential of breast cancer in relation to tumor size and age [10–12]. Through such models, it is possible to come up with an estimate of tumor size at an earlier stage in cases where diagnosis and treatment has been delayed. Contrary to this, there is no consensus on how to estimate the lymph node status at an earlier time even though the number of metastases is known at the time of treatment.

Several studies, among those a study from DBCG [13] and a study from Finland [14], have described mathematical models for predicting the risk of further LNM based on sentinel node (SN) status. Unfortunately, there is not a similar option for estimating lymph node status if the diagnosis was made earlier. An algorithm or a model could be a useful tool in evaluating the impact on treatment and the deterioration of prognosis as a consequence of delayed diagnosis (i.e. insurance cases). Isheden et al. [15] present, based on screening data, a mathematical model for the relation between the breast cancer tumor size and the probability of affected lymph nodes. Their model does not make it possible to accurately estimate the number of positive lymph nodes in the event of an advanced diagnosis but only provides a probability of whether there would have been LNM or not.

The purpose of this study is, by using large datasets from the DBCG database, to describe the association between the observed tumor size and the incidence of LNM and how this association depends on various patient data and tumor characteristics. Furthermore, it is hypothesized that for the individual tumor, the tendency for metastasizing follows a certain pattern. Tumors, which metastasize at an early stage, will have more metastases at a later stage than tumors of the same size, which have their first metastasis later. Furthermore, it is assumed that tumor characteristics do not change over time. Based on these assumptions, the aim is to describe a new model for predicting the occurrence of LNM at an earlier time for breast cancer patients where, at a given time, this association is known.

## Patients/material and methods

**Figure 1:**
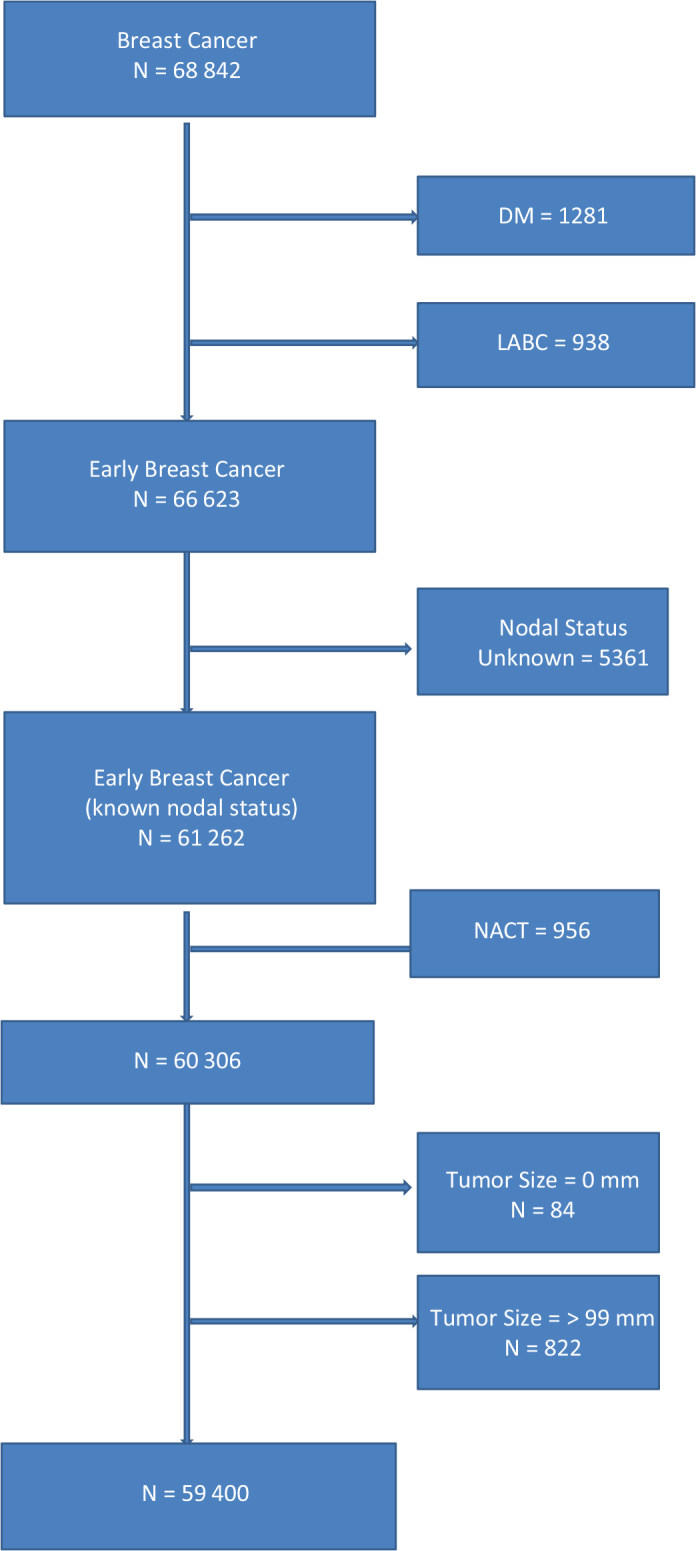
Study population. DM: Distant Metastasis; LABC: Locally Advanced Breast Cancer; Early Breast Cancer: population of women with breast cancer with no prior chemotherapy nor systemic treatment; Nodal Status: lymph node status; NACT: Neo Adjuvant Chemo Therapy.

DBCG, The Danish Breast Cancer Cooperative Group, was established in 1977. By using standard forms, data on breast cancer patients in Denmark have been collected prospectively since then. There were data on 68,842 breast cancer patients from the DBCG database over the years 1995–2012. Patients with prior neoadjuvant chemotherapy (NACT), with distant metastasis, locally advanced breast cancer (LABC), unknown nodal status, and patients with tumor size registered as 0 mm or larger than 99 mm were excluded. We thus included 59,400 patients who had primary surgery for early breast cancer (Figure 1 flow-chart).

The data from DBCG include age, time of treatment (year), menopausal status, tumor size (diameter), lymph node status (number of positive nodes), localization in breast, focality, histological type, grade, estrogen receptor (ER) status, lympho-vascular invasion (LVI), and type of surgery. HER2 receptor status was available for only 48% of the patients, as HER2 status was first implemented in Denmark in 2007.

The relationship between the tumor size and lymph node status is described. Tumor size was categorized in groups as follows: < 5 mm, 5–9 mm, 10–14 mm, 15–19 mm, 20–24 mm, 25–29 mm, 30–34 mm, 35–39 mm, 40–44 mm, 45–49 mm, and ≥ 50 mm. For each size group, the number of metastatic lymph nodes is given as percentiles.

## Statistics

Data are presented as proportions with percent in parentheses. Associations between various independent patient characteristics and tumor variables and LNM (yes/no) as the dependent predictors were analyzed through univariate and multivariate logistic regression and presented as odds ratios with 95% confidence intervals. For statistical analysis, the Stata 17 statistical package was used (StataCorp. 2021. Stata Statistical Software: Release 17. College Station, TX: StataCorp LLC).

Study type should be defined with reference to the relevant EQUATOR guideline.

## Results

The population of women with breast cancer over the years 1995–2012 was 68,842. After exclusions, the final cohort contained 59,400 patients, as depicted in Figure 1.

Descriptive data are presented in Table 1. The largest age group was 50–69 years at the time of surgery, constituting 57.4% of the population. Of the women in the population, 75% were postmenopausal and 54% were node negative. Regarding tumor size, the majority of women had a tumor size of 11–20 mm (42.1%). The most common histological type was ductal (83.2%), and most were grade II (38.4%). Three-quarters (76.7%) of the tumors were ER positive, and only 2.9% had unknown ER status. HER2 status was known in 28,496 patients (48.0%). Of those, 16.3% were HER2 positive. 80.8% of the tumors had no lympho-vascular-invasion (LVI). Most tumors were localized in the upper lateral quadrant (43.3%), and most tumors were unifocal (77.5%). A little more than half of the population were treated by mastectomy (51.5%).

**Table 1:** Demographic data.

Demographic data	*N*	%
**Age at operation**		
< 40	2,578	4.34
≥ 40–49	8,946	15.06
≥ 50–69	34,085	57.38
≥ 70–84	12,492	21.03
≥ 85	1,299	2.19
	59,400	100
**Menopause**		
Pre-menopausal	14,637	24.68
Post-menopausal	44,655	75.30
Unknown	108	0.02
	59,400	100
**Year of surgery**		
1995–2001	19,837	33.40
2002–2007	19,557	32.93
2008–2012	20,006	33.69
	59,400	100
**Tumor size (mm)**		
0–10	10,613	17.87
11–20	25,018	42.12
21–30	14,643	24.65
31–50	7,216	12.15
51+	1,910	3.22
	59,400	100
**Histological type**		
Ductal	49,424	83.21
Lobular	6,510	10.96
Other	3,141	5.29
Unknown	325	0.55
	59,400	100
**Grade (ductal and lobular, other/unknown)**		
I	16,327	27.49
II	22,788	38.36
III	12,070	20.32
Unknown	8,215	13.83
	59,400	100
**Estrogen receptor**		
Negative (0–9%)	12,080	20.34
Positive (10–100%)	45,586	76.74
Unknown	1,734	2.92
	59,400	100
**HER2 receptor**		
Normal	23,840	40.13
Positive	4,656	7.84
Unknown	30,904	52.03
	59,400	100
**Lymphovascular invasion**		
No	47,975	80.77
Yes	7,715	12.99
Unknown	3,710	6.25
	59,400	100
**Lymph node status**		
Node negative	32,093	54.03
Macrometastasis	22,762	38.32
Micrometastasis	4,545	7.65
	59,400	100
**Positive lymph nodes**		
0	32,096	54.03
1–3	17,821	30.00
4–9	5,937	9.99
10+	3,546	5.97
	59,400	100
**Localization of tumor in the breast**		
Upper lateral	25,736	43.33
Upper medial	8,102	13.64
Lower lateral	6,200	10.44
Lower medial	3,222	5.42
Central	5,345	9.00
Multiple localization/overlap	10,789	18.16
Unknown	6	0.01
	59,400	100
**Focality**		
Multifocal	6,129	10.32
Unifocal	46,023	77.48
Unknown	7,248	12.20
	59,400	100
**Surgery**		
Mastectomy	30,581	51.48
BCS	27,870	46.92
BCS and secondary mastectomy	949	1.60
	59,400	100

BCS: breast-conserving surgery.

Among the 59,400 patients in the present cohort, 27,307 (46.0%) were node positive. Among those, macro-metastasis (≥ 2 mm) was present in 38.3% and micro-metastasis (≥ 0.2 and < 2.0 mm) in only 7.7% (Table 2). The proportion of node positive tumors increased with increasing tumor size. Among the smallest tumors up to 5 mm in diameter, 18.0% had an LNM as opposed to 80.2% in tumors larger than 50 mm. The same pattern was not found for micrometastases. The proportion of women diagnosed with micro-metastasis was less than 10% in all tumor size groups up to 50 mm (Table 2).

**Table 2:** Tumor size and lymph node status. Material restricted to tumors < 100 mm.

Tumor size (mm)	Node negative	%	Macrometastases	%	Micrometastases	%	Total	%
< 5	1,106	81.99	141	10.45	102	7.56	1,349	100
5–9	4,988	82.07	711	11.70	379	6.24	6,078	100
10–14	8,153	68.97	2,660	22.50	1,008	8.53	11,821	100
15–19	7,045	56.80	4,232	34.12	1,127	9.09	12,404	100
20–24	4,703	47.48	4,372	44.14	830	8.38	9,905	100
25–29	2,522	42.27	2,995	50.19	450	7.54	5,967	100
30–34	1,594	37.92	2,350	55.90	260	6.18	4,204	100
35–39	678	30.89	1,388	63.23	129	5.88	2,195	100
40–44	539	27.70	1,295	66.55	112	5.76	1,946	100
45–49	235	27.68	575	67.73	39	4.59	849	100
> 50	530	19.76	2,043	76.17	109	4.06	2,682	100
Total	32,093	54.03	22,762	38.32	4,545	7.65	59,400	100

The occurrence of LNM was almost the same among ER positive and ER negative tumors, 45.7% and 47.5%, respectively, but there was a clear tendency toward more node positive patients with small tumors up to 15 mm among ER negative tumors, while ER positive tumors 15 mm or above in size had relatively more LNM (Supplement Figure 1).

The association between node positivity and age is depicted in Table 3. As shown in Table 3, there was a tendency toward a lower frequency of nodal disease in the age group 50–75 than both younger and older patients. A similar pattern was observed in regard to tumor size. There were smaller tumor sizes among the ER positive patients in the age group 50–69 years (Supplement Figure 2). In the cohort with known HER2 status, HER2 positive tumors represented 16.3%. HER2 positive tumors had more LNM: 56.9% versus 44.7% (OR 1.65 [95% CI 1.54–1.75]).

**Table 3:** Age and lymph node status.

Age	Node positive	%	Node negative	%	Total	%
< 40	1,388	53.84	1,190	46.16	2,578	4.34
40–49	4,545	50.80	4,401	49.20	8,946	15.06
50–69	14,928	43.80	19,157	56.20	34,085	57.38
70–84	5,768	46.17	6,724	53.83	12,492	21.03
≥ 85	675	51.96	624	48.04	1,299	2.19
	27,304		32,096		59,400	100

In univariate logistic regression analysis, significant associations were found between included clinical variables and tumor characteristics and the occurrence of node positivity (Table 4). In the multivariate model, most of the associations were confirmed, but for ER negativity, a discrepancy was discovered. The univariate analysis showed a higher risk of LNM in ER negative patients (OR 1.07 [95% CI 1.03–1.12]) as ER negative patients, in general, had larger tumors, while the opposite was found in the multivariate analysis (OR 0.74 [95% CI 0.71–0.78]). Furthermore, in the multivariate analysis, a positive correlation was observed for tumor size, higher grade, vascular invasion, and multifocality. A negative correlation was observed for ‘other’ histological types. Regarding the localization of the tumor in the breast, there was a significant difference in the occurrence of node positivity. Compared with tumors located in the upper lateral quadrant, centrally located tumors and tumors in the lower lateral quadrant more often were node positive, while in tumors located in the medial quadrants, fewer were detected with metastasis. It is also noticed that patients treated by breast conservation had nodal spread to a lower degree than seen in patients treated by mastectomy (OR 0.72 [95% CI 0.69–0.75]) (Table 4).

**Table 4:** Association between lymph node status and tumor characteristics.

Variable		Univariate logistic regression		Multivariate logistic regression	
		OR	95% CI	OR	95% CI
Tumor localization	Upper lateral	1		1	
	Upper medial	0.55	0.52–0.58	0.57	0.54–0.61
	Lower lateral	1.04	0.98–1.10	1.12	1.05–1.19
	Lower medial	0.77	0.72–0.83	0.88	0.81–0.95
	Central	1.94	1.83–2.06	1.35	1.26–1.45
	Multiple regions	1.01	0.96–1.05	0.98	0.93–1.03
	Unknown	2.29	0.42–12.48	1.87	0.32–10.93
Tumor size (mm)	Cont. variable	1.06	1.06–1.06	1.05	1.05–1.06
Age	Cont. variable	0.99	0.99–1.00	0.99	0.99–0.99
Surgery	Mastectomy	1		1	
	Lumpectomy	0.43	0.41–0.44	0.72	0.69–0.75
	Mastectomy after lumpectomy	0.92	0.81–1.05	0.99	0.85–1.14
Menopause	Pre-menopausal	1		1	
	Postmenopausal	0.79	0.76–0.82	0.99	0.94–1.06
	Unknown	0.70	0.22–2.21	0.79	0.20–3.07
Estrogen receptor (ER)	ER pos	1		1	
	ER neg	1.07	1.03–1.12	0.74	0.71–0.78
	Unknown	0.89	0.81–0.98	0.82	0.73–0.91
Histological type	Ductal	1		1	
	Lobular	0.98	0.93–1.03	0.97	0.91–1.03
	Other	0.38	0.35–0.42	0.74	0.65–0.83
	Unknown	0.88	0.71–1.10	1.39	1.08–1.80
Malignancy Grade	I	1		1	
	II	1.74	1.67–1.80	1.27	1.21–1.32
	III	2.04	1.95–2.14	1.19	1.12–1.25
	Unknown	0.82	0.78–0.87	0.66	0.60–0.71
Vascular invasion	None	1		1	
	Vascular invasion	7.56	7.10–8.05	5.16	4.84–5.52
	Unknown	1.26	1.18–1.35	1.35	1.23–1.48
Focality	Unifocal	1		1	
	Multifocal	1.96	1.85–2.07	1.44	1.36–1.54
	Unknown	0.84	0.80–0.83	0.84	0.79–0.90

OR: odds ratio; CI: confidence interval.

Univariate and multivariate logistic regression. HER2 status not included in the analysis.

*N* = 59,400.

In multiple logistical regression including the HER2 status, the associations found in the total cohort were confirmed (Supplementary Table 1 and Supplementary Table 2). Furthermore, it was shown that HER2 positive tumors had a slightly increased occurrence of node positivity (OR 1.17 [95% CI 1.09–1.26]).

Finally, the association between subtypes based on ER and HER2 was explored in a separate model restricted to patients with tumors where both receptor-values were known (Table 5). In this analysis, compared to ER positive and HER2 negative tumors, a strong negative association between the ER negative and HER2 negative subtype and node positivity was noticed (OR 0.57 [95% CI 0.52–0.63]).

**Table 5:** Association between lymph node status and tumor characteristics.

Variable		Multivariate logistic regression	
		OR	95% CI
Tumor localization	Upper lateral	1	
	Upper medial	0.57	0.52–0.63
	Lower lateral	1.06	0.97–1.17
	Lower medial	0.97	0.86–1.09
	Central	1.28	1.15–1.42
	Multiple regions	0.89	0.83–0.95
	Unknown	-	-
Tumor size (mm)	Cont. variable	1.06	1.05–1.06
Age	Cont. variable	0.99	0.98–0.99
Surgery	Mastectomy	1	
	Lumpectomy	0.63	0.59–0.67
	Mastectomy after lumpectomy	1.03	0.83–1.26
Menopause	Pre-menopausal	1	
	Postmenopausal	0.95	0.87–1.04
	Unknown	0.56	0.08–3.80
Histological type	Ductal	1	
	Lobular	0.79	0.72–0.86
	Other	0.88	0.72–1.07
	Unknown	1.25	0.82–1.92
Malignancy Grade	I	1	
	II	1.21	1.13–1.29
	III	1.10	1.00–1.20
	Unknown	0.58	0.49–0.68
Vascular invasion	None	1	
	Vascular invasion	5.92	5.34–6.56
	Unknown	1.59	1.29–1.95
Focality	Unifocal	1	
	Multifocal	1.30	1.18–1.42
	Unknown	0.74	0.68–0.80
Subtype	ER pos, HER2 neg	1	
	ER pos, HER2 pos	0.97	0.89–1.07
	ER neg, HER2 pos	0.96	0.86–1.08
	ER neg, HER2 neg	0.57	0.52–0.63

ER: estrogen receptor; OR: odds ratio; CI: confidence interval.

Univariate and multivariate logistic regression for patients with known ER and HER2 status. Stratified for subtypes.

*N* = 28,298.

From both analysis (Tables 4 and 5), the most striking observation was the increased odds for LVI (OR 5.16 [95% CI 4.84–5.52] and OR 5.92 [95% CI 5.34–6.56], respectively).

In Table 6, the occurrence of LNM (including both micro- and macro-metastasis) stratified by the tumor size is further described and presented as percentiles. As it appears, for tumors up to 19 mm, the median rate of nodal metastasis is 0. For tumors in the range 20–34 mm, the median is 1 and 2 for tumors from 35 to 49 mm. For tumors larger than 50 mm, the median value was 4. Similar information for the individual subtypes is given in the supplementary material (Supplementary Tables 3–6).

**Table 6:** Number of metastatic lymph nodes stratified by tumor size.

Tumor diameter (mm)	Percentiles								
	10	20	30	40	50	60	70	80	90
**< 5**	0	0	0	0	0	0	0	1	1
**5–9**	0	0	0	0	0	0	0	0	1
**10–14**	0	0	0	0	0	0	1	1	2
**15–19**	0	0	0	0	0	1	1	2	4
**20–24**	0	0	0	0	1	1	2	3	6
**25–29**	0	0	0	0	1	1	2	4	8
**30–34**	0	0	0	1	1	2	3	5	10
**35–39**	0	0	0	1	2	2	4	7	12
**40–44**	0	0	1	1	2	3	5	8	14
**45–49**	0	0	1	1	2	4	6	9	14
**> 50**	0	1	1	2	4	6	9	12	17

Both micro- and macrometastases included.

*N* = 59,400.

The model for the estimation of lymph node status at an earlier stage is based on the tabulated percentiles. It is a prerequisite that for a given tumor, during growth, the relationship between the size of tumor and the number of LNM will fall within the same percentile. The correlated values can therefore be read directly in Table 6 (see also Appendix A).

## Discussion and conclusion

Detailed information about the factors that determine the incidence of LNM in early breast cancer is given in this study, comprising almost 60 000 patients, of whom 46% had LNM. In summary, the tumor size turned out to be a major discriminating factor for nodal involvement; for each increase in the tumor diameter of 1 mm, the proportion of patients with LNM increased by 5%. In general, the risk of LNM increased with more aggressive tumor characteristics, and most significantly for LVI, which increased the risk of LNM by more than 5 times. Patients with ER negative and HER2 negative tumors, however, stood out with a decreased proportion of LNM compared with other subtypes. A significant observation was also an association between the risk of nodal spread and the localization of the tumor in the breast, with fewer LNM if the tumor was located medially in the breast and more LNM in centrally located tumors. Finally, the risk of LNM was also found to be lower after breast-conserving surgery (BCS).

The strength of this study lies in its huge cohort and the large number of descriptive variables. Since 1977, data from Danish breast cancer patients have been registered prospectively using standard forms. Furthermore, all citizens in Denmark have their own Personal Civil Registration Number, which enables DBCG to incorporate data, increasing the quality of the registry data. DBCG presents data with a high degree of completeness, minimizing the risk of bias due to missing variables.

The limitations of this study are few. Only 48% of this cohort has HER2 status registered as HER2 was introduced in 2007 in Danish breast cancer tumor profiling. Despite the missing variables, this study shows a substantial number of patients with HER2 registration, allowing valid and conclusive data. This data set is from 1995 to 2012, and we are not concerned that the results will have changed significantly since then. We believe that the associations and conclusions drawn from these data will be the same despite the fact that treatment might have changed over time.

The most important prognostic factor in breast cancer patients is LNM. This study has confirmed that the tumor size is an important factor for the risk of developing axillary metastasis. The larger the tumor, the higher the risk of axillary metastasis.

Sopik et al. [16] examined 819,647 women from the database Surveillance, Epidemiology and End Results (SEER) and also found a correlation between the increasing tumor size and LNM at the time of diagnosis. They reported on tumor sizes between 1 and 150 mm at the time of diagnosis and found that the risk of LNM increased proportionally to tumor size. They found that 32.3% were lymph node positive at the time of diagnosis (among patients with known lymph node status, 264,027) as opposed to 46% in our study. Unlike our study, Sopik et al. [16] included macrometastases only as they identified lymph nodes ≤ 200 individual cells or ≤ 0.2 mm as N0/node negative. In our study, 38.3% of the 46% had macrometastases.

There are similarities between this study and the study by Sopik et al. [16] Among tumors < 10 mm, there were 11.5% node positive in our study. In the SEER population, the proportion was around 11%. We cannot rule out that this present study reports on too many small tumors with LNM. Even though we have excluded patients with a tumor size of 0 mm, it could be that some ‘occult breast cancers’ diagnosed because of the LNM has been registered with small tumor sizes < 5 mm.

Tumor size is measurable and predictable in growth over time as shown in prior studies [10, 12]. Development in lymph node status, on the other hand, is less predictable. There are several published studies, where models for predicting a general risk for LNM based on tumor size, tumor biology, and patient characteristics are presented [1, 9, 15, 17]. Even though such studies can estimate the risk of developing LNMs over time, they cannot be used for estimating the individual patient´s risk of having metastases at an earlier time, which is very relevant for evaluating the consequence of delayed diagnosis and treatment of breast cancer in insurance cases.

Based on data from the present study, it is possible in a rather simple way to estimate the nodal status at an earlier time in patients where diagnosis and treatment has been delayed. See a description of the model in Appendix A. Such a model must, of course, be used with caution when starting from an individual patient. However, the fact that it is possible to distinguish between different tumor characteristics (ER positive-HER2 negative, ER positive - HER2 positive, ER negative - HER2 postitive, and ER negative - HER2 negative) makes it possible to adapt the model more precisely to the individual situation (Appendix A). Still, one has to be aware that the model depends on the ability to calculate the tumors’ growth.

Our results also confirm what is already known from several studies including Greer et al. [9] and Malter et al. [18] that LVI is a strong independent predictor for LNM. Furthermore, the present study indicates that the risk of detecting LNM depends on the localization of the tumor in the breast. Thus, patients with tumors located in the upper medial quadrant were less often diagnosed with LNM, whereas patients with centrally located tumors were more often diagnosed with metastases. Bevalicqua et al. [19, 20], Yoshira et al. [1], and Lohrisch et al. [21] have reported similar findings. They all point to an alternative lymphatic route, leading to the parasternal lymph nodes as an explanation. This might indicate that some LNM is under-detected in these patients, and thereby these patients are wrongly stated and undertreated. This goes well with their findings of a higher mortality among these patients. We are not able to add further evidence to that, as we have no survival data, but if their observations can be confirmed, which we will try to do in a future study, then it might be relevant to include tumor localization into the treatment algorithms for axillary node negative patients with breast cancer.

ER negative and HER2 negative patients had a much lower risk of LNM than other subtypes. The data also showed that HER2 positivity was an independent risk factor for LNM tumors, although it was rather weak (increased risk 17%). Very similar results were found by Holm-Rasmussen et al. [8] who examined more than 20,000 women diagnosed with primary breast cancer. Women with both micro-metastasis and macro-metastasis were classified as lymph node positive in their study. Holm-Rasmussen [8] also looked at the subtypes based on ER and HER2 receptors and like us found a significantly decreased risk of LNM in patients with ER negative and HER2 negative tumors, but, unlike us, they also found an increased risk in ER negative and HER2 positive tumors. We are not able to explain why the results are somewhat different in the two studies, other than it might be related to different population sizes.

We found that patients undergoing BCS versus mastectomy had a lower risk of LNM (OR 0.72 [95% CI 0.69–0.75]). This might indicate that in some patients who undergo BCS, LNMs are missed at the operation. The access to the axilla is more difficult when performed together with BCS than in connection with mastectomy. Therefore, it is, at least in theory, possible that smaller LNMs are not detected in relation to axillary exploration through axillary clearance or SN biopsy. On the other hand, if this happens, it seems not to influence the prognosis. Several studies, including a large DBCG study [22], have shown that the survival after BCS is at least as good if not better than after mastectomy.

Tumor size is a major determinant for LNM in breast cancer, but there is no lower limit of tumor size for LNM. The risk of LNM is increased in more aggressive tumors and most significantly for LVI, which increases the risk by more than five times. Patients with ER negative and HER2 negative tumors have less often LNM compared to other subtypes. Based on our data, we present a model for estimating nodal status in patients where diagnosis and treatment has been delayed. Such estimates should though be interpreted with caution in the individual case, as the tumor growth rate may vary.

## Supplementary Material



## Data Availability

Data are not publicly available due to Danish law.
