# Supplementary material for: The association between lymph node status and the tumor size in breast cancer – results from the Danish Breast Cancer Group (DBCG)
Source: Acta Oncol. 2025 Aug 5;64:43380. doi: 10.2340/1651-226X.2025.43380 (PMC12340993; doi:10.2340/1651-226X.2025.43380)
Supplement: Supplementary file 1 [file AO-64-43380-s1.pdf]

Supplementary material has been published as submitted. It has not been copyedited, or typeset by Acta Oncologica

Supplementary Table 1. Association between lymph node status and tumor characteristics.

**Variable**		**Multivariate logistic regression**	
		**OR**	**95% CI**
Tumor localization	Upper lateral	1	
	Upper medial	0.58	0.54-0.61
	Lower lateral	1.12	1.06-1.19
	Lower medial	0.87	0.80-0.95
	Central	1.32	1.23-1.41
	Multiple regions	0.94	0.90-0.99
	Unknown	1.98	0.34-11.54
Tumor size (mm)	Cont. variable	1.05	1.05-1.05
Age	Cont. variable	0.99	0.99-0.99
Surgery	Mastectomy	1	
	Lumpectomy	0.68	0.65-0.71
	Mastectomy after lumpectomy	0.96	0.83-1.10
Menopause	Pre-menopausal	1	
	Postmenopausal	0.99	0.94-1.05
	Unknown	0.77	0.20-3.07
Estrogen receptor (ER)	ER pos	1	
	ER neg	0.74	0.71-0.78
	Unknown	0.88	0.78-0.98
HER2 receptor	HER2 normal	1	
	HER2 positive	1.17	1.09-1.26
	Unknown	0.80	0.76-0.83
Histological type	Ductal	1	
	Lobular	0.96	0.89-1.02
	Other	0.70	0.62-0.79
	Unknown	1.33	1.03-1.72
Malignancy Grade	I	1	
	II	1.24	1.19-1.30
	III	1.14	1.07-1.21
	Unknown	0.68	0.63-0.74
Vascular invasion	None	1	
	Vascular invasion	5.17	4.84-5.53
	Unknown	1.53	1.39-1.68
Focality	Unifocal	1	
	Multifocal	1.41	1.33-1.50
	Unknown	0.78	0.73-0.84

Multivariate logistic regression including HER2 status.

N=54,304.

Supplementary Table 2. Association between lymph node status and tumor characteristics.

**Variable**		**Multivariate logistic regression**	
		**OR**	**95% CI**
Tumor localization	Upper lateral	1	
	Upper medial	0.57	0.52-0.62
	Lower lateral	1.07	0.97-1.17
	Lower medial	0.97	0.86-1.09
	Central	1.29	1.16-1.43
	Multiple regions	0.89	0.83-0.95
Tumor size (mm)	Cont. variable	1.05	1.05-1.06
Age	Cont. variable	0.99	0.98-0.99
Surgery	Mastectomy	1	
	Lumpectomy	0.63	0.59-0.67
	Mastectomy after lumpectomy	1.03	0.84-1.26
Menopause	Pre-menopausal	1	
	Postmenopausal	0.96	0.88-1.04
	Unknown	0.60	0.09-4.09
Estrogen receptor (ER)	ER pos	1	
	ER neg	0.68	0.63-0.74
HER2 receptor	HER2 normal	1	
	HER2 positive	1.17	1.09-1.27
Histological type	Ductal	1	
	Lobular	0.79	0.73-0.87
	Other	0.84	0.69-1.02
	Unknown	1.22	0.80-1.87
Malignancy Grade	I	1	
	II	1.19	1.12-1.27
	III	1.05	0.96-1.15
	Unknown	0.58	0.50-0.68
Vascular invasion	None	1	
	Vascular invasion	5.96	5.38-6.61
	Unknown	1.60	1.31-1.95
Focality	Unifocal	1	
	Multifocal	1.30	1.19-1.43
	Unknown	0.74	0.69-0.80

Multivariate logistic regression for patients with known HER2 status.

N=28,406.

Supplementary Table 3. ER negative and HER2 negative.

**Tumor size (mm)**	**Percentiles** ER negative HER2 negative								
	**10**	**20**	**30**	**40**	**50**	**60**	**70**	**80**	**90**
**<5**	0	0	0	0	0	0	0	1	1,5
**5-9**	0	0	0	0	0	0	0	1	1
**10-14**	0	0	0	0	0	0	0	1	2
**15-19**	0	0	0	0	0	0	1	2	4
**20-24**	0	0	0	0	0	1	1	2	4
**25-29**	0	0	0	0	0	1	2	3	8
**30-34**	0	0	0	0	1	1	2	4	8
**35-39**	0	0	0	0	1	1	3	5	10
**40-44**	0	0	0	1	1	3	4	7	14
**45-49**	0	0	0	1	1	2	3	5	10
**>50**	0	0	0	1	2	3	6	10	16

Supplementary Table 4. ER positive and HER2 negative.

Tumor size (mm)	Percentiles ER positive HER2 negative								
	**10**	**20**	**30**	**40**	**50**	**60**	**70**	**80**	**90**
**<5**	0	0	0	0	0	0	0	1	1
**5-9**	0	0	0	0	0	0	0	0	1
**10-14**	0	0	0	0	0	0	1	1	2
**15-19**	0	0	0	0	0	1	1	2	3
**20-24**	0	0	0	0	0	1	2	3	6
**25-29**	0	0	0	1	1	1	2	4	7
**30-34**	0	0	0	1	1	2	3	5	9
**35-39**	0	0	1	2	2	3	5	7,5	13,5
**40-44**	0	0	1	2	2	3	5	8	13
**45-49**	0	0	1	2	3	4	7	9	13
**>50**	0	1	2	3	4	6	9	12	17

Supplementary Table 5. ER positive and HER2 positive.

**Tumor size (mm)**	**Percentiles** ER positive HER2 positive								
	**10**	**20**	**30**	**40**	**50**	**60**	**70**	**80**	**90**
**<5**	0	0	0	0	0	0	0	0	1
**5-9**	0	0	0	0	0	0	0	1	1
**10-14**	0	0	0	0	0	0	1	1	3
**15-19**	0	0	0	0	1	1	1	3	7
**20-24**	0	0	0	1	1	1	2	4	8
**25-29**	0	0	0	1	1,5	2	3	6	10,5
**30-34**	0	0	1	1	2	3	5	8	13
**35-39**	0	0	1	1	2	3	5	7	10
**40-44**	0	1	1	3	4	6	8	12	17
**45-49**	0	0	0,5	1	2	3	4	8	18,5
**>50**	1	1	3	4	6	8	10	14	22

Supplementary Table 6. ER negative and HER2 positive.

**Tumor size (mm)**	**Percentiles**<br>ER negative HER2 positive								
	**10**	**20**	**30**	**40**	**50**	**60**	**70**	**80**	**90**
**<5**	0	0	0	0	0	0	1	2	4
**5-9**	0	0	0	0	0	0	1	2	4
**10-14**	0	0	0	0	0	1	1	2	5
**15-19**	0	0	0	0	1	1	2	4	8
**20-24**	0	0	0	1	1	2	3	7	12
**25-29**	0	0	0	1	1	2	4	6	11
**30-34**	0	0	1	1	2	3	7	8	16
**35-39**	0	0	0,5	1	2	3,5	6	8	10,5
**40-44**	0	1	2	2	4	5	8	13,5	18
**45-49**	0	0	1	2	3	4	8	8	14
**>50**	0	1	3	4	6	7	9	13	17

Supplementary Fig. 1 Lymph node status according to tumor size.

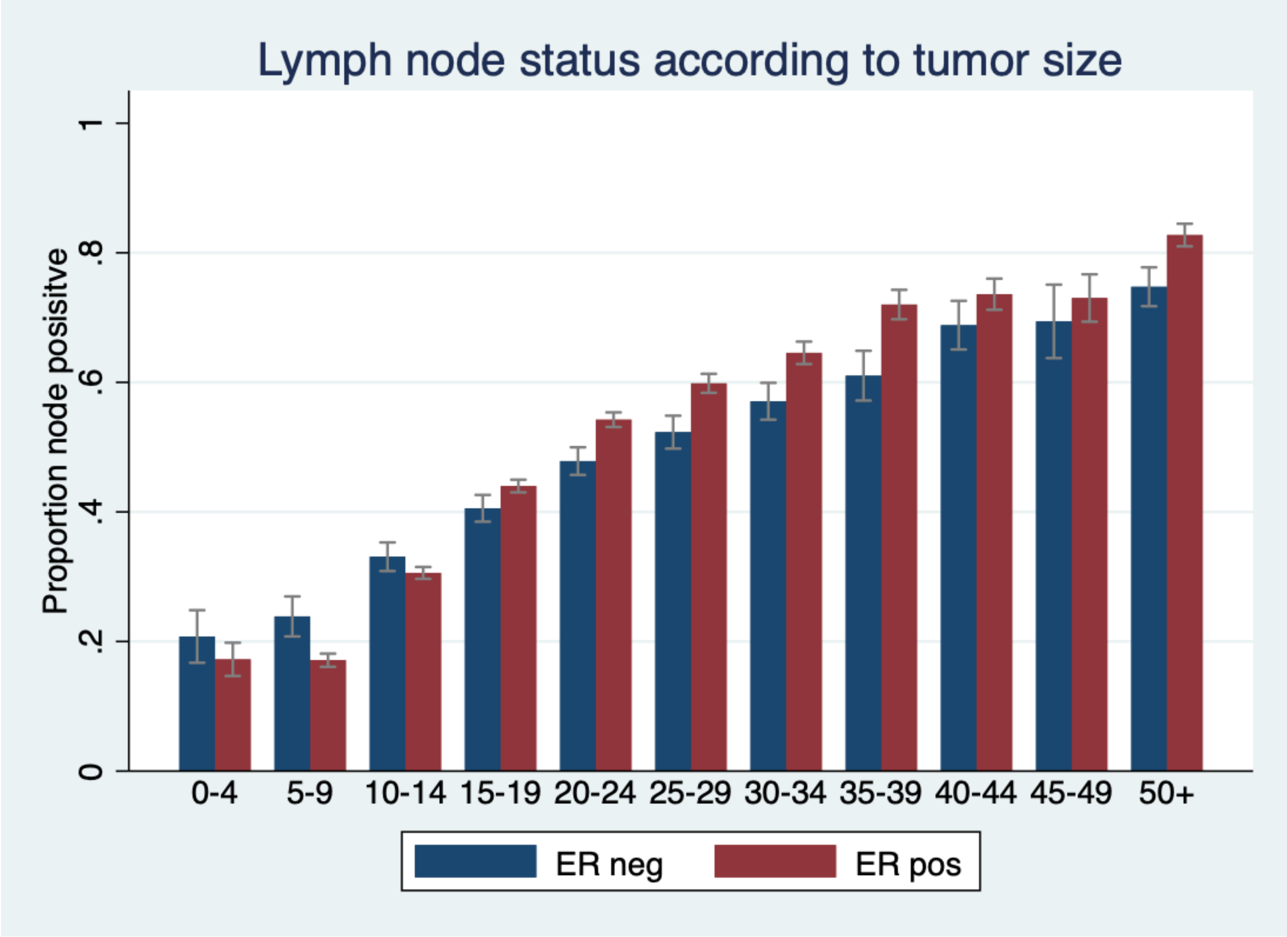

Supplementary Fig. 2 Tumor size according to age.

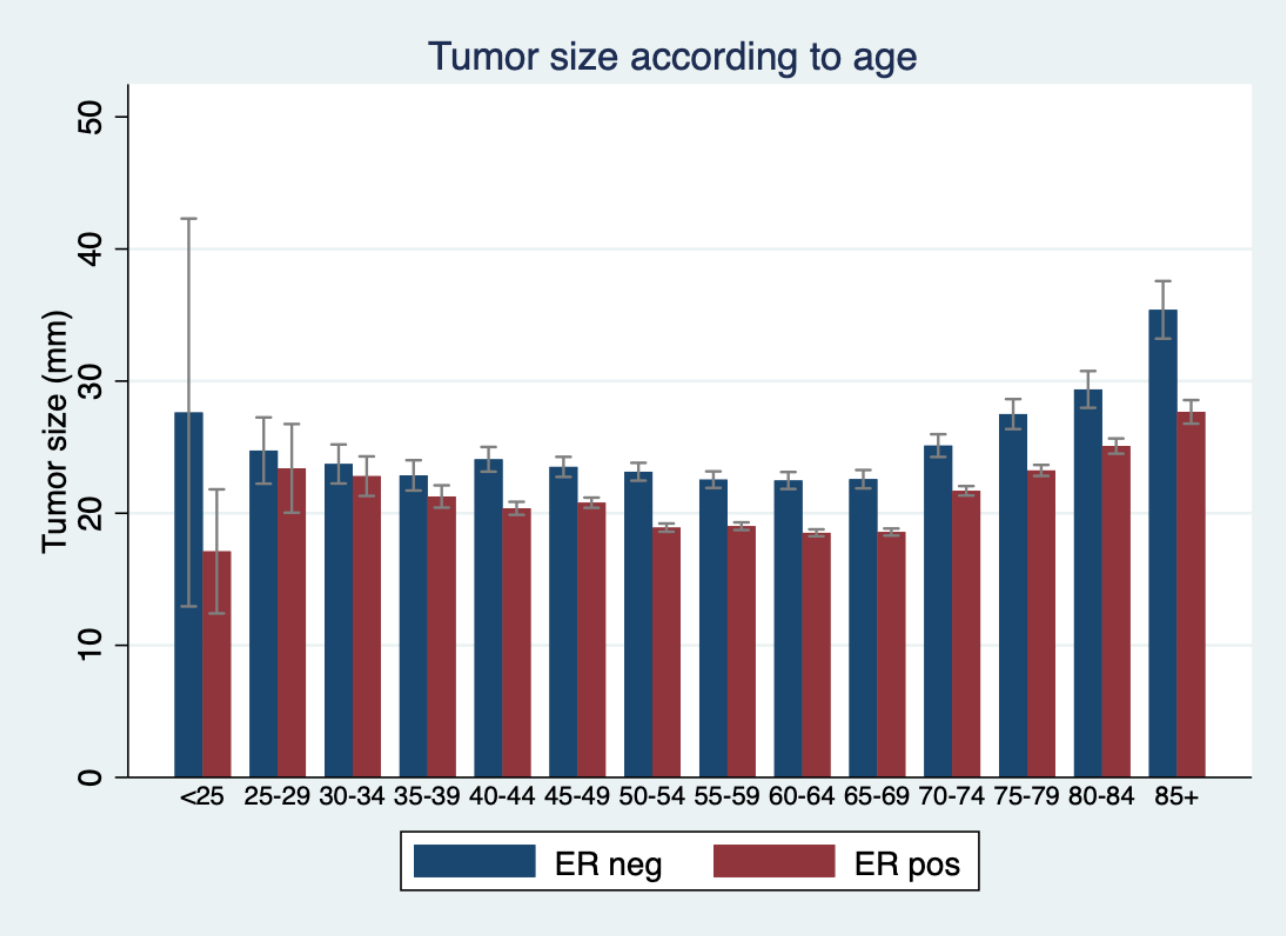

Appendix A

There are models for predicting the consequences of delayed diagnosis and treatment of breast cancer. Such models are able to specify how the tumor has grown during the delay, based on exponential growth rate [12] and assumptions on the doubling time (DT) for the tumor cells. DT has been evaluated by different imaging models, mostly mammography and ultrasound (US). In Peer et al.,[10] the growth rate was determined on the basis of mammography, and in their study, DT was found to be lower in premenopausal women than in postmenopausal women: 80 days (95 % CI 44-147) and 157 (95 % CI 121-204), respectively. In a recent publication by Nakashima et al.[23] based on US data, it was described that around 30 % of tumors showed no sign of growth within the examining period (interval mean 57 days). For the rest, DT was 174 days (interquartile range 97-360 days), and it was possible to further stratify DT by subtypes, with the lowest value for HER2 positive, ER negative of 85 days and the highest for ER positive, HER2 negative of 185 days. In this study most patients were postmenopausal, and there was no stratification on menopausal status. Thus, there seems to be no greater discrepancy between the reported DT from the two studies.

There are no available models which can predict the development of lymph node metastases (LNM) as a consequence of a delay in diagnosis and treatment, but based on the description of the relationship between tumor size and lymph node status in the present study, we suggest a simple model for that.

In the present algorithm for estimating the number of lymph node metastases by advancing diagnosis, it is assumed that for the individual tumor, the incidence of lymph node metastases will follow a certain pattern, corresponding to the distribution shown in Table 6. Thus, for a given tumor, during growth the relationship between size and number of LNM, will lie in the same interval delimited by percentiles.

As an example, for a tumor with a size of 30-34 mm with 3 LNM (70 percentile), the number of lymph node metastases is estimated to be 1, if it had been diagnosed at an earlier point in time, with a diameter lying in the range 15-19 mm, and if the tumor diameter was less than 10 mm at the time of diagnosis there would have been no LNM.

More precise estimations can be done, if the subtype is taken into consideration and the supplementary Tables 3-6 are used.
